# Assessment of plaque evolution in coronary bifurcations located beyond everolimus eluting scaffolds: serial intravascular ultrasound virtual histology study

**DOI:** 10.1186/1476-7120-11-25

**Published:** 2013-07-20

**Authors:** Il Soo Lee, Christos V Bourantas, Takashi Muramatsu, Bill D Gogas, Jung Ho Heo, Roberto Diletti, Vasim Farooq, Yaojun Zhang, Yoshinobu Onuma, Patrick W Serruys, Hector M Garcia-Garcia

**Affiliations:** 1 Thoraxcenter, Erasmus Medical Center, ‘s-Gravendijkwal 230, 3015 CE, Rotterdam, The Netherlands

**Keywords:** Bifurcation, Intravascular ultrasound-virtual histology, Plaque type, Coronary bifurcation, Intravascular ultrasound, Coronary artery disease, Atherosclerosis

## Abstract

**Purpose:**

To evaluate the atherosclerotic evolution in coronary bifurcations located proximally and distally to a bioresorbable scaffold.

**Methods:**

Thirty bifurcations located >5 mm beyond the scaffolded segment, being investigated with serial intravascular ultrasound virtual histology (IVUS-VH) examinations, at baseline and 2-years, in patients enrolled in the ABSORB cohort B1 study were included in this analysis. In each bifurcation, the frames portraying the proximal rim, in-bifurcation, and distal rim of the ostium of the side branch were analyzed. The geometric parameters and plaque types were evaluated at baseline and 2-years follow-up.

**Results:**

There were no significant differences in the geometrical parameters such as lumen, vessel and plaque areas as well as in the composition of the atheroma between baseline and 2-years follow-up.

When we separately examined the bifurcations located proximally and distally to the scaffolded segment, no changes were found at the distal bifurcations, while at the proximal bifurcations there was a statistical significant decrease in the plaque burden (36.67 ± 13.33% at baseline vs. 35.06 ± 13.20% at 2 years follow-up, p = 0.04).

Ten necrotic core rich plaques were found at baseline, of which 2 regressed to either fibrotic plaque or to intimal thickening at 2 years follow-up. The other 8 did not change. Disease progression was noted in 3 plaques (1 adaptive intimal thickening, 1 fibrotic and 1 fibrocalcific plaque) that evolved to necrotic rich plaques.

**Conclusions:**

Plaque regression was noted at the bifurcations located proximally to the bioresorbable scaffold but not at these located distally. Additional studies are required to confirm this finding and examine further the effect of drug elution on atherosclerotic evolution.

## Introduction

Although atherosclerosis is a systemic disease it has focal and eccentric manifestations. This paradox has been attributed to local triggers such as the blood flow hemodynamics [1,2]. Coronary bifurcations are prone to atherosclerosis as in these segments complex flow pattern are noted that can induce plaque development. Hence, lesions in coronary bifurcations are a common finding and in SYNTAX study 63% of the randomized patients had obstructive disease in these anatomies [3]. Coronary angiography has limited capability in assessing atherosclerotic disease progression/regression in bifurcation lesions as in these segments there is an overlapping of branches and the increased density of the contrast agent does not permit accurate quantification of vessel dimensions [4,5]. In addition, coronary angiography cannot provide any information regarding the plaque burden and quantify changes in its composition.

These drawbacks were addressed by intravascular ultrasound (IVUS) imaging which allows accurate quantification of the lumen and vessel wall dimensions and permits accurate assessment of atheroma burden [6]. The ability of gray-scale IVUS to characterize the composition of the coronary plaque appears to be moderate, a limitation that was overcome with the development of IVUS virtual histology (VH) that is able to provide accurate detection and quantification of different plaque types [7,8]. These unique qualities have rendered IVUS/IVUS-VH a useful tool in the study of atherosclerosis. Therefore many IVUS based trials have been conducted or are underway that examine the natural evolution of atherosclerosis and the effects of different invasive and non-invasive treatments on plaque progression [9-13].

With regards to coronary bifurcations there is only one report which used serial IVUS and optical coherence tomography (OCT) examinations to evaluate plaque progression [14]. In this report there was no difference in luminal dimensions, plaque composition and its burden between baseline and at 6 months follow-up. The reported results might be due to the short time interval between the 2 examinations. The aim of this study is to provide further information about the plaque evolution in coronary bifurcations. In contrast to the previous study the time interval between the baseline and follow-up examination is longer (2 years) and we analyze separately bifurcations located proximally and distally to an implanted drug eluting scaffold in order to detect a potential effect of the downstream drug delivery on atherosclerotic evolution.

## Methods

### Study population

**Figure 1:**
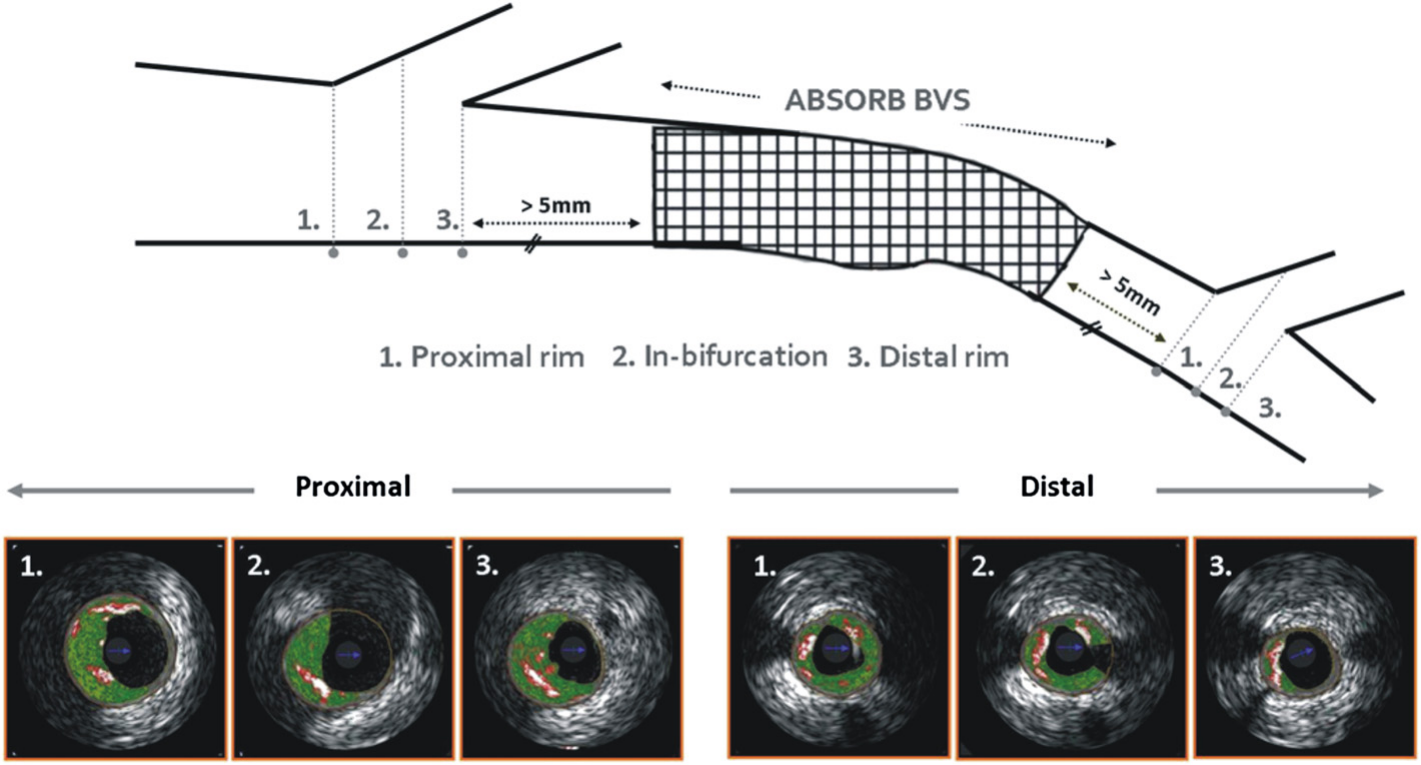
**Selection of the bifurcations included in the current analysis.** Only the bifurcations located >5 mm proximally and distally to the implanted scaffold were studied. Plaques were analyzed only in the main coronary artery at the proximal rim of the ostium of the side branch, at the in-bifurcation site and at the distal rim of the ostium of the side branch.

We analyzed data from patients with obstructive coronary artery diseases who underwent percutaneous coronary intervention with the everolimus-eluting bioresorbable vascular scaffold (BVS; ABSORB®, Abbott Vascular, Santa Clara, CA, USA). Out 45 included patients that were enrolled in the ABSORB cohort B1 study and had invasive assessment including IVUS and IVUS-VH examinations at baseline and at 2 years follow-up [15], 12 patients portrayed bifurcations located >5 mm away from the scaffold (19 proximally and 11 distally) segment and had side branch orifice measured by IVUS >1.5 mm (Figure 1). The sponsor of this study was Abbott Vascular.

### IVUS-VH acquisition

IVUS examination was performed by a phased-array, 20MHz, 3.2F IVUS catheter (Eagle Eye, Volcano Corporation, Rancho Cordova, California) using an automatic continuous pull-back device operating at a speed of 0.5 mm/s. The IVUS grayscale and IVUS-VH analyses were performed offline using the pcVH 2.1 software (Volcano Corporation, Rancho Cordova, California).

### Bifurcation analysis

**Figure 2:**
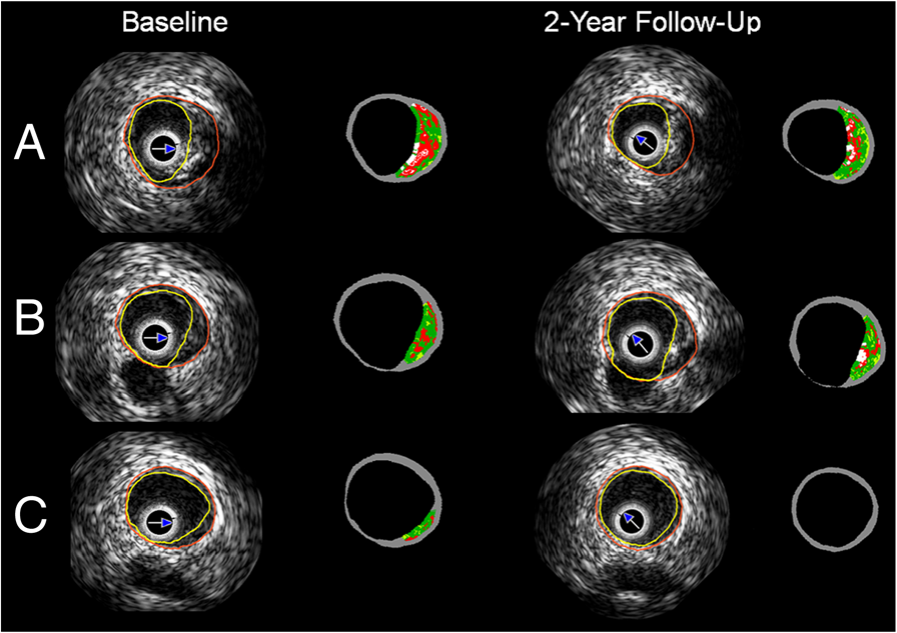
**A typical example of an analyzed bifurcation segment at baseline and at 2 years follow-up.** Panel **A** portrays the proximal rim of the ostium of the side branch, **B** the in-bifurcation segment and **C** the distal rim of the ostium of the side branch.

Only the pullback in the main branch was available for the current analysis. The analyzed bifurcation lesions were classified into the proximal or distal bifurcation group based on its location. The proximal bifurcation group included bifurcations located proximally to the scaffolded segment and the distal group these located distally. In each bifurcation the cross-sectional frames that portrayed 1) the proximal rim of the ostium of the side branch, 2) the bifurcation cross-section and 3) the distal rim of the ostium of the side branch, were identified and included in the final analysis. The proximal rim of the ostium of the side branch was portrayed by the first frame proximally to the take off of the side branch; the bifurcation cross-section corresponded to the frame with the largest ostial diameter of the side branch while the distal rim of the ostium of the side branch by the first frame located distally to the side branch (Figure 2). In each IVUS frame the lumen area, vessel area and plaque area were measured while the plaque burden was computed by the equation: plaque burden = 100 x plaque area/vessel area [6].

### Plaque type classification

IVUS-VH allowed identification of four plaque types: dense calcium (DC), necrotic core (NC), fibrofatty (FF) and fibrotic tissue (FT) that are illustrated in a color coded map, with the white color corresponding to the calcified tissue, the red to the necrotic core, the light green to the fibrofatty and the green to fibrotic tissue. Based on the type of the plaque detected by IVUS-VH, 2 experienced observers classify the atheromas in 2 groups: 1) those with high percentages of fibrotic tissue without a significant amount of NC (adaptive intimal thickening, pathological intimal thickening, fibrocalcific plaque, and fibrotic plaque); and 2) those that had an increased plaque burden (>40%) and more than 10% NC component (fibroatheroma and calcified fibroatheroma). In case that the NC was in direct contact with the lumen the atheroma was classified as thin cap fibroatheroma (TCFA) or calcified thin cap fibroatheroma when the Ca burden was >10% [16,17].

These plaque types were reported per location within the bifurcation (i.e., cross-section at the proximal rim of the ostium of the side branch, at the bifurcation point and at the distal rim of the ostium of the side branch) and per bifurcation using a hierarchical approach that classified the atheroma based on the worst plaque type detected within the 3 studied frames [18]. To evaluate changes in the composition of the plaque which may have a potential impact on prognosis, we estimated an IVUS-VH-derived plaque risk index in each bifurcation. This indice has already been described in the previous literature [14], and is given as the ratio of the sum of the NC-rich plaques vs. the sum of non-NC-rich plaques.

### Statistical analysis

Categorical variables are presented as frequencies and percentages. Continuous variables are presented as means ± standard deviations. For 2-related sample comparison of continuous variables the Wilcoxon test was used. A p value <0.05 was considered statistically significant. The bifurcation (lesion) was the unit of analysis without corrections for correlated observations in the same subjects. Statistical analyses were performed using SPSS version 18.0 for Windows (SPSS, Inc., Chicago, IL, USA).

## Results

### Baseline characteristics

The median age of the studied population was 65.52 ± 8.01 years; 60% of the patients had stable angina and all of them were treated with statins during the follow-up. None of these patients had sustained a cardiovascular event due to disease progression in the studied bifurcation. The clinical characteristics of the 12 patients (30 bifurcations, 19 located proximally and 11 distally to the BVS) included in the analysis are illustrated in Table 1.

**Table 1:** Baseline clinical and angiographic characteristics of the studied population (n = 12)

**Patients characteristics**	**Number of patients**
Age	65.52 ± 8.01
Men	9 (75%)
Hypertension	4 (33%)
Hypercholesterolemia	11 (92%)
Diabetes	0 (0%)
Smoking	0 (0%)
Previous MI	4 (33%)
Previous PCI	2 (17%)
*Clinical presentation*	
Stable angina	10 (83%)
Unstable angina	1 (8%)
Silent ischemia	0 (0%)
**Treated vessel**	
LAD	7 (58%)
LCX	3 (25%)
RCA	2 (17%)
**Medications**	
Beta-blockers	6 (50%)
ACE inhibitors/ARBs	5 (41%)
Statins	12 (100%)
Calcium-channel blocker	3 (25%)

Table footnote: *ACE* angiotensin-converting enzyme, *ARB* angiotensin receptor blocker, *PCI* percutaneous coronary artery, *LAD* left anterior descending coronary artery, *LCX* left circumflex coronary artery, RCA right coronary artery.

### Geometrical and compositional analysis of the IVUS derived measurements

Table 2 provides the results from the geometrical and compositional analysis at the proximal rim of the ostium of the side branch, in-bifurcation, and distal rim of the ostium of the side branch in all analyzed bifurcations. The lumen, vessel wall areas and the plaque burden did not change at 2 years follow-up. No statistical significant differences were noted in the composition of the plaque between baseline and follow-up apart from a decrease in the fibrotic tissue component at the proximal rim of the ostium of the side branch (1.15 mm^2^ vs. 0.70 mm^2^, p = 0.01).

**Table 2:** Luminal vessel wall and plaque measurements and compositional data in each region of the bifurcation at baseline and at 2 years follow-up (n = 30 bifurcations)

**Variable**	**Baseline**	**2 years follow-up**	**p value**
**Vessel CSA (mm**^**2**^**)**			
Distal	12.94 ± 4.35	13.22 ± 4.36	0.27
In-bifurcation	14.96 ± 5.65	15.11 ± 5.78	0.33
Proximal	15.22 ± 5.99	15.23 ± 5.89	0.80
**Lumen CSA (mm**^**2**^**)**			
Distal	7.89 ± 3.16	8.16 ± 2.80	0.20
In-bifurcation	10.02 ± 4.35	10.25 ± 4.40	0.28
Proximal	9.05 ± 4.06	9.34 ± 4.25	0.25
**Plaque CSA (mm**^**2**^**)**			
Distal	5.05 ± 3.19	5.05 ± 3.36	0.58
In-bifurcation	4.93 ± 2.93	4.90 ± 3.17	0.98
Proximal	6.18 ± 3.48	5.87 ± 3.33	0.12
**Plaque burden (%)**			
Distal	37.54 ± 14.21	36.36 ± 14.20	0.23
In-bifurcation	32.63 ± 12.60	31.74 ± 12.71	0.41
Proximal	39.43 ± 13.70	38.11 ± 14.35	0.25
**DC CSA (mm**^**2**^**)**			
Distal	0.37 ± 0.80	0.33 ± 0.63	0.84
In-bifurcation	0.28 ± 0.55	0.32 ± 0.59	0.25
Proximal	0.30 ± 0.50	0.30 ± 0.51	0.46
**DC (%)**			
Distal	11.86 ± 21.49	9.40 ± 13.28	0.93
In-bifurcation	15.61 ± 22.30	14.04 ± 18.14	0.86
Proximal	10.66 ± 18.39	9.84 ± 15.56	0.87
**NC CSA (mm**^**2**^**)**			
Distal	0.42 ± 0.72	0.49 ± 0.90	0.44
In-bifurcation	0.38 ± 0.58	0.43 ± 0.82	0.48
Proximal	0.51 ± 0.71	0.55 ± 0.79	0.84
**NC (%)**			
Distal	11.00 ± 13.18	12.94 ± 16.25	0.51
In-bifurcation	13.63 ± 11.04	15.66 ± 12.83	0.36
Proximal	13.69 ± 11.17	15.08 ± 13.05	0.31
**Fibrofatty CSA (mm**^**2**^**)**			
Distal	0.19 ± 0.37	0.26 ± 0.76	0.97
In-bifurcation	0.32 ± 0.69	0.28 ± 0.75	0.23
Proximal	0.32 ± 0.52	0.32 ± 0.74	0.53
**Fibrofatty tissue (%)**			
Distal	5.41 ± 8.65	6.32 ± 15.03	0.85
In-bifurcation	8.42 ± 12.82	6.48 ± 11.42	0.39
Proximal	8.68 ± 10.25	9.09 ± 13.95	0.60
**Fibrotic CSA (mm**^**2**^**)**			
Distal	0.94 ± 1.41	0.78 ± 1.25	0.26
In-bifurcation	1.05 ± 1.27	0.93 ± 1.32	0.26
Proximal	1.62 ± 1.70	1.21 ± 1.43	0.01
**Fibrotic tissue (%)**			
Distal	28.39 ± 30.79	21.38 ± 24.45	0.13
In-bifurcation	35.44 ± 27.72	37.20 ± 26.75	0.82
Proximal	46.28 ± 28.32	38.33 ± 28.77	0.10

Table footnote: *CSA* cross sectional area, *DC* dense calcium, *NC* necrotic core.

### Distribution of IVUS-VH-derived plaque types at the three bifurcation regions

The distribution of NC-rich and NC-poor plaques is presented in Table 3. NC-rich plaques were more frequently located at proximal rim and distal rim than the in-bifurcation site at baseline and at 2 years follow-up. The plaque risk index was higher at 2 years follow-up suggesting increase of the number of NC-rich plaques.

**Table 3:** Plaque type at distal rim, in- bifurcation, and proximal rim of the ostium of the side branch at baseline and at 2 years follow-up and IVUS-VH derived plaque risk index

	**Non-NC-rich plaques**				**NC-rich plaques**				
**Segment**	**AIT**	**PIT**	**FC**	**FT**	**CaFA**	**FA**	**CaTCFA**	**TCFA**	Plaque risk index*
Distal rim,BL	18 (60)	1 (3.3)	3 (10)	2 (6.7)	4 (13.3)	1 (3.3)	1 (3.3)	0 (0)	6/24 = 0.25
Distal rim, 2y	19 (63.3)	0 (0)	3 (10)	1 (3.3)	6 (20)	0 (0)	1 (3.3)	0 (0)	7/23 = 0.30
In- bifurcation, BL	21 (70)	0 (0)	5 (16.7)	1 (3.3)	2 (6.7)	1 (3.3)	0 (0)	0 (0)	3/27 = 0.11
In- bifurcation, 2y	24 (80)	0 (0)	4 (13.3)	0 (0)	2 (6.7)	0 (0)	0 (0)	0 (0)	2/28 = 0.07
Proximal rim, BL	17 (56.7)	1 (3.3)	3 (10)	3 (10)	2 (6.7)	0 (0)	2 (6.7)	1 (3.3)	5/24 = 0.21
Proximal rim, 2y	17 (58.6)	1 (3.4)	2 (6.9)	2 (6.9)	4 (13.8)	0 (0)	3 (10.3)	0 (0)	7/22 = 0.32

Table footnote: *AIT* adaptive intimal thickening, *BL* baseline, *CaFA* calcified fibroatheroma, *CaTCFA* calcified thin-cap fibroatheroma, *FA* fibroatheroma, *FC* fibrocalcific plaque, *FT* fibrotic plaque, *IVUS* intravascular ultrasound, *NC* necrotic core, *PIT* pathologic intimal thickening, *TCFA* thin-cap fibroatheroma, *VH* virtual histology.

* The plaque risk index is defined as the sum of NC rich vs. non-NC rich plaques.

### Geometric and compositional comparison of the IVUS measurements based on the location of the bifurcation

**Figure 3:**
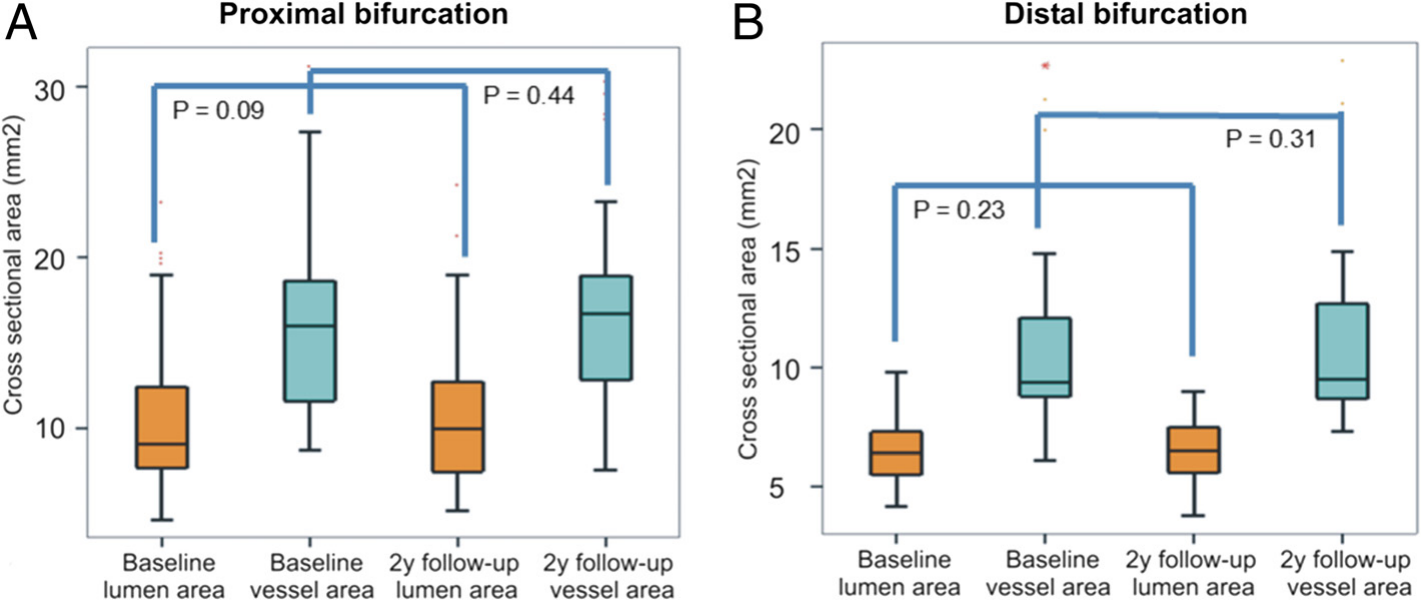
Lumen and vessel wall areas at baseline and at 2 years follow-up at the bifurcations located proximally (A) and distally (B) to the bioresorbable scaffold (proximal and distal bifurcation group).

**Figure 4:**
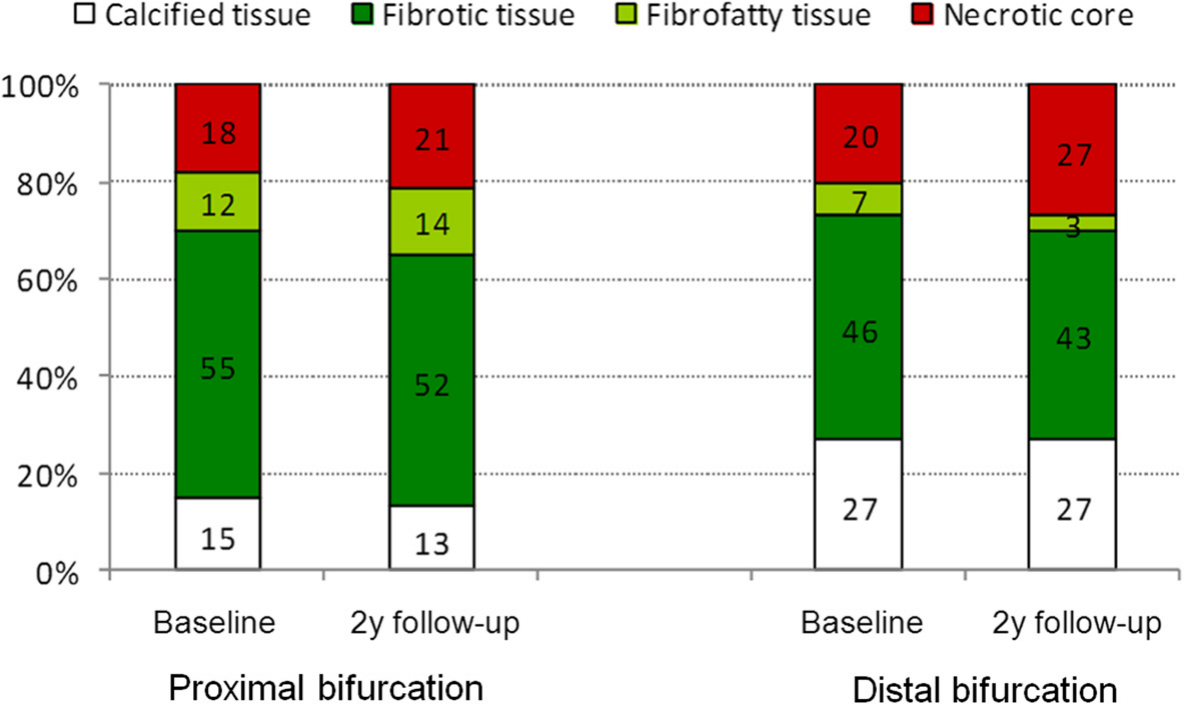
**Plaque composition at the bifurcations located proximally and distally to the bioresorbable scaffold (proximal and distal bifurcation group) at baseline and at 2 years follow-up.** The white color corresponds to dense calcium, the red to necrotic core, the light green to fibrofatty tissue and the dark green to fibrotic tissue.

As it would have been expected the vessel and lumen cross-sectional areas were larger at the proximal bifurcations than at the distal bifurcations at baseline and at 2 years follow-up (p < 0.05). There were no changes in the lumen and vessel areas in both proximal (10.38 ± 4.23 mm^2^ vs. 10.67 ± 4.14 mm^2^, p = 0.09 and 16.37 ± 5.15 mm^2^ vs. 16.50 ± 5.20 mm^2^, p = 0.44 respectively) and distal group (6.62 ± 1.67 mm^2^ vs. 6.83 ± 1.86 mm^2^, p = 0.23 and 10.96 ± 3.98 mm^2^ vs. 11.13 ± 3.88 mm^2^, p = 0.31, respectively) between baseline and 2 years follow-up (Figure 3). On the other hand at follow-up the percent plaque burden was decreased at the proximal bifurcation from 36.67 ± 13.33% to 35.06 ± 13.20% (p = 0.04), but did not change at the distal bifurcation (36.22 ± 14.44% vs. 35.91 ± 15.15%, p = 0.67). There were no changes in the composition of the plaque in the proximal group (p = 0.633 for the DC p = 0.58 for the NC, p = 0.84 for the fibrofatty and p = 0.10 for the fibrotic component) while at the distal group there was only a reduction in the fibrofatty component at 2 years follow-up (p = 0.73 for the DC, p = 0.13 for the NC, p = 0.02 for the fibrofatty and p = 0.53 for the fibrotic tissue) (Figure 4).

### Bifurcation plaque type

**Figure 5:**
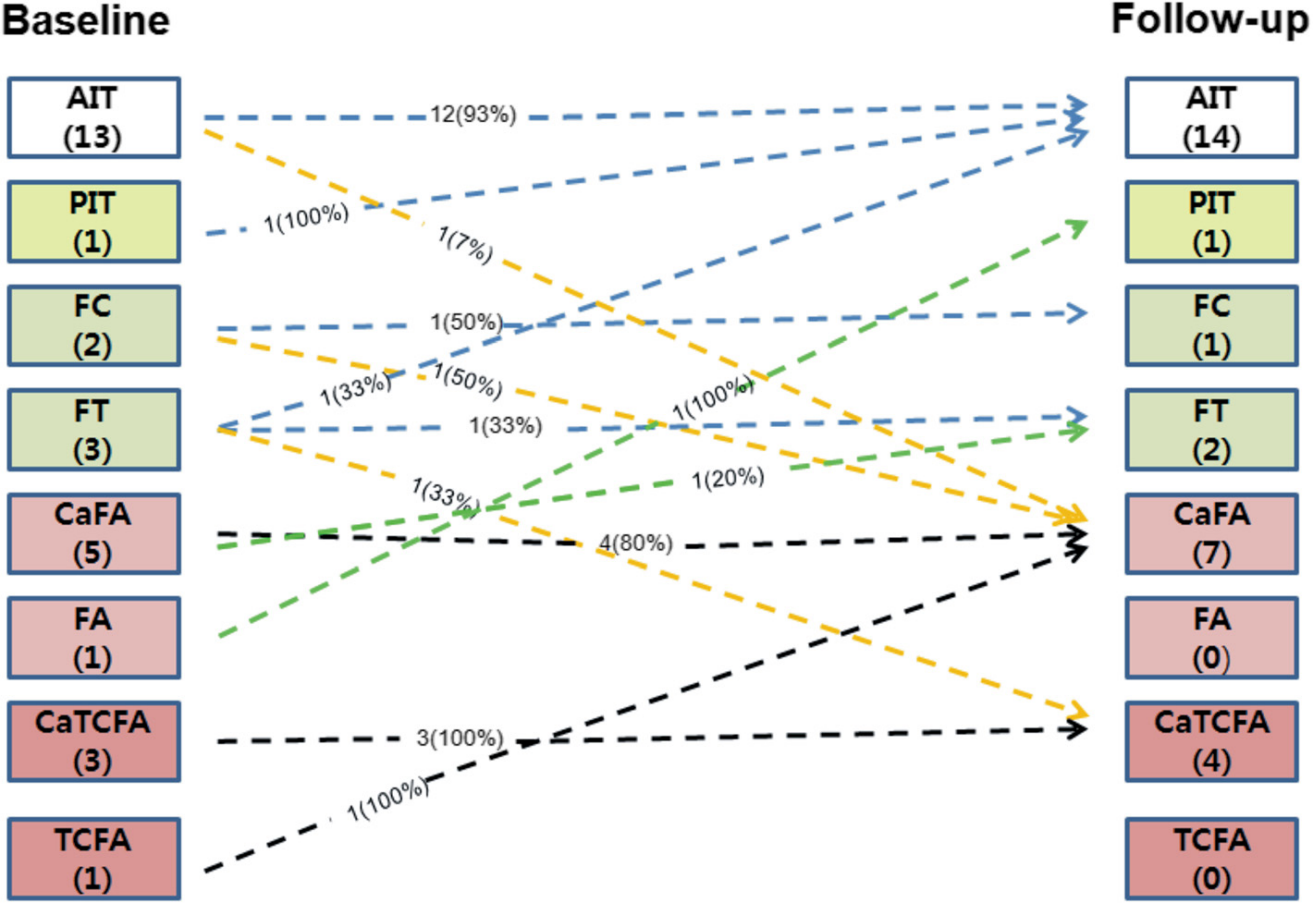
**Changes in type of the plaque type detected at the coronary bifurcations.** For each plaque type, the percent of changes is reported. AIT, adaptive intimal thickening; CaFA, calcified fibroatheroma; CaTCFA, calcified thin-cap fibroatheroma; FA, fibroatheroma; FC, fibrocalcific plaque; FT, fibrotic plaque; PIT, pathologic intimal thickening; TCFA, thin-cap fibroatheroma.

At baseline, 10 NC-rich plaques were found, 4 of which were classified as thin-cap lesions (1 TCFA and 3 calcified TCFA) (Figure 5). One fibroatheroma became pathological intimal thickening and another calcified fibroatheroma became fibrotic plaque suggesting disease regression in these plaques. On the other hand 3 new NC-rich lesions developed from 1 adaptive intimal thickening, 1 fibrotic and 1 fibrocalcific plaques implying disease progression. Eight NC-rich plaques (80%) did not change at follow-up. Most of the thin-cap lesions (n = 3, 75%) were also unchanged apart from 1 calcified TCFA that became calcified fibroatheroma.

## Discussion

In this analysis we used serial IVUS-VH to examine the atherosclerotic evolution in coronary bifurcations. We found no significant changes with respects to the luminal and vessel wall dimensions, and plaque burden as well as its composition at 2 years follow-up. However, when we examined separately the bifurcations located proximally and distally to an implanted BVS, we noted plaque regression in proximal bifurcation group while the plaque burden did not change in the distal group.

Although there is sufficient evidence to support the prognostic implications of the atheroma burden and its composition, there is limited information about the atherosclerotic evolution [12,19,20]. In a recent report Kubo et al. used serial IVUS-VH to study changes in the type of the plaque and the atheroma burden in 99 patients. Two hundred sixteen non-culprit lesions were evaluated at baseline and at 12 months follow-up. At follow-up 12 TFCAs developed while most (75%) of the detected TCFAs (n = 20) at baseline evolved to a more stable plaque types suggesting disease regression [9]. This study highlighted the dynamic nature of coronary atherosclerosis and demonstrated that atherosclerotic evolution includes not only disease progression but it is also likely high risk plaque to regress and become more stable. In a similar study Diletti et al. used serial OCT and IVUS-VH examinations to assess changes in the atheroma burden and its composition in coronary bifurcations [14]. Twenty seven NC-rich plaques were detected at baseline most of which (81%) did not show any significant change at 6 months follow-up. Although the bifurcation lesions analyzed in the present study revealed lower plaque risk index, our results are similar to this latter report. We found no differences in the plaque volume and its composition but we also reported regional changes of the type of the plaque as 2-rich NC plaques regressed to more stable forms and 3 new NC-rich plaques developed at follow-up. An increase in the plaque risk index was noted at 2 years suggesting disease progression even under the standard medication therapy including statins.

It is well known that bifurcation anatomies are prone to atherosclerosis; and recently we have showed that the lesions located at the bifurcation site have an increased atheroma burden and lipid component compared to the lesions located in a non-bifurcated segment [21]. The differences in the plaque composition and burden between bifurcation and non-bifurcation lesions should at least partially be attributed to the complex flow patterns seen in these segments. The local hemodynamics seems to affect not only the plaque burden but also the distribution of the plaque which is unevenly distributed in coronary bifurcations. In a Gonzalo et al. showed that NC-rich plaques are seen more often in the proximal rim of the ostium of the side branch than in the distal rim [16]. We also found an increased lipid component and plaque burden in the proximal rim of the ostium of the side branch and in the distal rim of the ostium of the side branch while less plaque was seen in the in-bifurcation segment. The increased plaque burden seen in the proximal rim of the ostium of the side branch should be probably attributed to the disturbed flow often seen before the flow divider [22]. On the other hand in the carina the shear stress is high and thus the plaque burden was found to be reduced ^10^. At the distal rim of the ostium of the side branch and in particular at the outer side of the flow divider low or oscillating shear stress have been detected and these segments appear to be susceptible to atherosclerosis [23]. Although the number of the bifurcations studied is limited there was a difference in the evolution of the plaque in the proximal and distal bifurcation group. In particular the plaque burden appeared to be reduced in the proximal bifurcation while there were no changes in the distal. The reduction of the plaque burden in proximal bifurcation can be attributed to the treatment with statins [24,25]. Although the small number of the distal bifurcations studied does not allow us to draw firm conclusions and potentially detect significant changes between baseline and follow-up the effect of the downstream drug delivery from the BVS on vessel wall physiology and on the distal bifurcation cannot also be excluded. Several studies have demonstrated that implantation of a drug eluting stent can affect plaque development [26,27]. Krasouski et al. demonstrated that patients treated with a drug eluting were less likely to develop stenoses at the downstream vessel and events compared to patients receiving bare-metal stents fact that was attributed to a beneficial effect of the downstream drug delivery [26]. On the other hand, Wakabayashi et al. showed that in patients treated with a paclitaxel eluting stent there is an increase in the plaque burden up to 15 mm distally to the implanted stent. This has been attributed to the inflammation triggered by the antiproliferative drug [27]. In addition, cumulative evidence suggests that after drug-eluting stent implantation there is endothelial dysfunction in the distal vessel due to the eluted drug which can promote plaque progression [28-30]. Although there is lack of evidence about the impact of everolimus elution on endothelial function and on plaque development in the downstream vessel a reaction of the vessel to the presence of the drug cannot be excluded. This could potentially promote atherosclerosis and hence counterbalance the effect of statins.

### Study limitations

The most important limitation of this analysis is the small number of the recruited patients and the limited number of the bifurcations studied. Hence this report may be underpowered to detect differences in plaque burden and composition. Thus this study should be considered as an exploratory and hypothesis generating analysis and the results should be interpreted with caution. In view of the limited data we were restricted either to a descriptive report or to a simplified statistic analysis and we did not take into consideration the clustering effect. Furthermore, in contrast to previous studies serial IVUS imaging was performed only in the main branch and not in the side branch of the bifurcation lesion [31]. This is a significant limitation of our study since we were not able to evaluate coronary pathology in side branches and detect changes in plaque composition and burden in these segments.

## Conclusions

This study analyzed serial IVUS-VH data obtained at baseline and 2 years follow-up from patients with obstructive coronary artery disease implanted with a BVS and treated with statins and demonstrated no significant differences in the plaque burden or its composition at coronary bifurcations located more than 5 mm from the treated segment. Analyzing separately bifurcations located proximally and distally to the BVS we found plaque regression at the proximal group and no changes in the plaque at the distal group. This may potentially be due to the effect of the downstream drug delivery on vessel wall physiology; however further studies are required to confirm this finding and examine the consequences of the everolimus elution on the downstream vessel.

## Competing interest

The authors declare that they have no competing interest.

## Authors’ contribution

ISL carried out mainly to study this article. I designed this study with Hector, collected all data from ABSORB cohort B1,and drafted the manuscript. CVB carried out collection of statistics of our study and drafted the manuscript correctly. He deeply participated this study. TM also carried out collection of statistics, made figures and participated writing manuscript. BDG Participated making figures and commented how to study this article. JHH also designed this study, carried out collection of statistics and deeply commented how to study continuously. RD commented how to study this article and participated making table. VF commented making tables and how to study. YZ carried out collection of statistics and made figures. YO commented how to study and encouraged me continuously. PWS is great professor. He taught me how to study this article continuously. He also encouraged, supervised me every time. HMG is Direct of our research labs. He designed this article, supervised me all. He finally decided to submit this manuscript to Cardiovascular Ultrasound. All authors read and approved the final manuscript.
